# Association between human herpes simplex virus and severe headache or migraine among aged 20–49 years: a cross-sectional study

**DOI:** 10.3389/fneur.2024.1476863

**Published:** 2024-10-08

**Authors:** Tao Zheng, Li Jiang, Guanglu Li, Na Zeng, Binyang Yu, Shaojie Duan, Gesheng Wang, Zunjing Liu

**Affiliations:** ^1^Beijing University of Chinese Medicine, Beijing, China; ^2^Department of Brain Disease III, Dongfang Hospital Beijing University of Chinese Medicine, Beijing, China; ^3^Shaodong People's Hospital, Hunan, China; ^4^Department of Geriatrics, Taizhou Central Hospital (Taizhou University Hospital), Zhejiang, China; ^5^Department of Neurology, Peking University People’s Hospital, Beijing, China

**Keywords:** herpes simplex virus, severe headache, migraine, cross-sectional study, NHANES

## Abstract

**Background and purpose:**

Previous studies have shown that human herpes simplex virus (HSV) infection may be associated with the onset of headache or migraine. We aimed to investigate the association between HSV infection and severe headache or migraine.

**Materials and methods:**

The cross-sectional data on 5,730 participants aged 20–49 years were obtained from the 1999–2004 National Health and Nutrition Examination Survey (NHANES). We used weighted logistic regression analysis to assess the association between HSV infection (HSV-1 gG-1 and HSV-2 gG-2) and severe headache or migraine, and performed subgroup analyses.

**Results:**

Our study found that women, higher education, higher body mass index, better family conditions, smoking and alcohol consumption were all associated with severe headaches or migraines. After adjusting for confounding factors such as sex, age, race, and education, HSV-2 (+) was still significantly associated with severe headache or migraine (OR = 1.22, 95%CI:1.03–1.46, *p* = 0.0443). In subgroup analyses, we found that participants with HSV-1 (−) and HSV-2 (+) were also significantly associated with severe headache or migraine (OR = 1.41, 95%CI:1.04–1.91, *p* = 0.0281).

**Conclusion:**

HSV-2 gG-2(+) was significantly associated with severe headache or migraine.

## 1 Introduction

Epidemiological studies show that 52% of people worldwide suffer from general headache, migraine, tension headache and other headache diseases every year (1). As a neurological disorder with a high risk of disability, migraine can directly affect more than 1 billion people worldwide (2). And migraine is the leading cause of disability in women under the age of 50 years (3). The pathogenesis of headache is complicated and the etiology is varied. Several studies have found that certain infectious inflammatory diseases are associated with an increased risk of migraine (4, 5). Napier et al. found that herpes simplex virus (HSV) infection may be related to the pathogenesis of migraine (6). In addition, research by Meineri et al. found that 42% of patients with new daily persistent headache were infected with HSV (7). However, additional research is needed to establish a more definitive relationship between HSV infection and headache or migraine.

HSV is a widely transmitted virus that is often associated with neurological diseases such as herpes simplex encephalitis (HSE) and neonatal herpes. The trigeminal theory is one of the main theories to explain the pathogenesis of migraine (8). The theory is that the onset of migraine involves the activation of the trigeminal vascular system. Specifically, pain production in migraine is associated with neurons in the trigeminal ganglion, which project onto the meninges and emit central axons to reach trigeminal vascular neurons in the spinal nucleus of the trigeminal nerve, leading to peripheral sensitization of the migraine. The axons of secondary trigeminal vascular neurons further project pain signals to the brain stem, hypothalamus, basal ganglia, and multiple nuclei in the thalamus, forming central sensitization. HSV-1 can be latent in the trigeminal ganglion, and when the body’s immune function is reduced, the virus is activated and retrograde along the central branch of the trigeminal nerve to the base of the brain, causing acute encephalitis, usually limited to the temporal and frontal lobes of the orbital part (9). The pathogenesis of neonatal HSV infection involves the virus entering the host through sensory nerve endings, transporting to the posterior root ganglion along the direction of the inverse nerve axon, and incubating there for life (10). It can be seen that HSV can be latent in ganglia through nerve fibers and cause disease when the virus reactivates. We aimed to investigate the link between HSV infection and severe headache or migraine, and to explore the underlying mechanism.

National Health and Nutrition Examination Survey (NHANES) database provides health information on a large population, including epidemiological data on headaches and HSV infections. We conducted a cross-sectional study using the 1999–2004 NHANES database to explore the association between HSV infection and severe headache or migraine.

## 2 Methods

### 2.1 Study population

**Figure 1:**
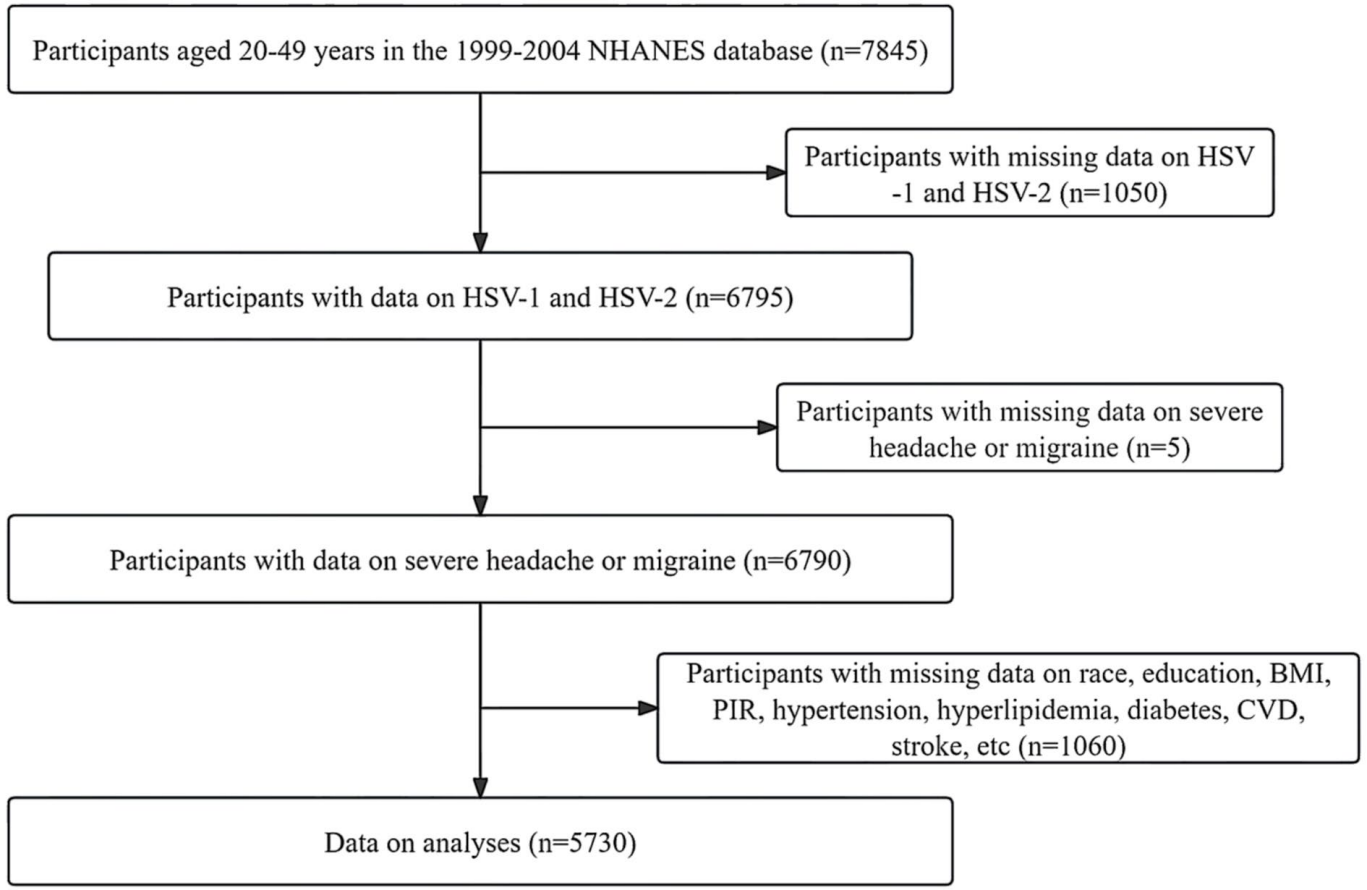
Detailed information about participants recruitment. HSV, human simple virus; BMI, body mass index; CVD, cardiovascular events; PIR, poverty to income ratio.

This study is based on a publicly available NHANES database from 1999 to 2004. The purpose of the NHANES project is to assess the health and nutrition status of deinstitutionalized Americans using stratified multistage probabilistic surveys. NHANES collects demographic and in-depth health information through home visits, screenings, and laboratory tests conducted by mobile screening centers (MECs). Based on available data on HSV and severe headache or migraine, 6,790 participants aged 20–49 years were initially included. After we excluded missing or inactive participants for poverty-to-income ratio (PIR), education level, hypertension, diabetes, the body mass index (BMI), total cholesterol (TC), high-density lipoprotein cholesterol (HDL-C), cardiovascular events (CVD), stroke, and smoking status, 5,730 participants remained. Due to the lack of data on alcohol consumption, we used the “multiple interpolation method” to interpolate the above missing data (11, 12). Detailed information about participant recruitment is shown in Figure 1.

### 2.2 HSV infection

According to previous NHANES-based research methods, the determination of HSV infection mainly relies on the detection of HSV-1 and HSV-2 antibodies in participants’ serum by specific glycoproteins G (HSV-1 gG-1 and HSV-2 gG-2). Serum reactive to an immunodot charged with gG-1 indicates the person being tested has HSV-1 infection [HSV-1 (+)]. Serum reactive to an immunodot charged with gG-2 indicates the person being tested has HSV-2 infection [HSV-2 (+)]. According to the HSV infection status of the enrolled participants, they were divided into six types: HSV-1 (+), HSV-2 (+), HSV-1 (−) and HSV-2 (−), HSV-1 (+) and HSV-2 (−), HSV-1 (−) and HSV-2 (+), HSV-1 (+) and HSV-2 (+) (13).

### 2.3 Definition of severe headache or migraine

Based on the Pain Questionnaire of 1999–2004 NHANES, we defined severe headaches or migraines as: “Have you experienced severe headaches or migraines in the past 3 months?”

### 2.4 Other covariates

The selection of covariates is based on existing literature and biological factors (13–15). Demographic characteristics include age, sex, education level, race or ethnicity, PIR, smoking status, alcohol consumption, cardiovascular events, stroke, etc. The mean systolic blood pressure (SBP), mean diastolic blood pressure (DBP), and BMI were selected for each participant during the physical examination. In laboratory tests, TC, HDL-C and glycosylated hemoglobin (HBA1c) were selected. We divided education levels into three groups: below high school, high school, and above high school. Race and ethnicity were grouped into five categories: Mexican American, non-Hispanic black, non-Hispanic white, other Hispanic, and other racial groups (14, 15). We classified household income based on PIR as low (PIR ≤ 1.3), medium (1.3 < PIR < 1.85), and high (PIR ≥ 1.85) (15). The smoking status of our participants was divided into three groups: non-smokers, current smokers, and former smokers. Participants who smoked <100 cigarettes in their lifetime were defined as never smoking. We defined previous smoking as having smoked >100 cigarettes in a lifetime, but no longer smoking. Current smoking was defined as smoking >100 cigarettes sometimes or daily in a lifetime. BMI was divided into three subgroups: BMI < 25 kg/m^2^, 25 kg/m^2^ ≤ BMI < 30 kg/m^2^, and BMI ≥ 30 kg/m^2^ (13). In addition, we defined drinking as “Had at least 12 alcohol drinks per year.” Diabetes is defined as having been told by a doctor that they have diabetes, or having an HbA1c > 6.5%. Hypertension is defined as having been told by a doctor that he or she has high blood pressure, or that SBP ≥ 140 mmHg, or DBP ≥ 90 mmHg. Hyperlipidemia is defined as TC > 5.18 mmol/L, or HDL-C < 1.04 mmol/L in men and < 1.3 mmol/L in women. CVD includes self-reported coronary atherosclerotic heart disease, congestive heart failure, heart attack, and angina pectoris.

### 2.5 Statistical analyses

We calculated and combined the weights for 1999–2002 and 2003–2004 according to the NHANES analysis Guide. Categorical data is expressed as frequency and weighted percentage, while continuous variables are expressed as mean (standard deviation, SD). For baseline features, we used t-tests or non-parametric tests to analyze the statistical difference of continuous variables, and chi-square tests to analyze the statistical difference of categorical variables. We used weighted univariate and multivariate logistic regression analysis to study the relationship between different HSV infection status and migraine. The regression model was tested by gradually adjusting for potential confounding factors (Models 1–3). Model 1 is a crude model without adjusting any covariates. Model 2 was adjusted for sex, age, race, education level, PIR, and BMI. Model 3 was based on model 2, and then adjusted for confounding factors such as smoking status, alcohol consumption, hypertension, hyperlipidemia, diabetes, CVD and stroke. *p* < 0.05 was statistically significant. This study is based on the statistical analysis of DecisionLink.1.0.^1^

## 3 Results

### 3.1 Baseline characteristics

The baseline information of all participants in this study is shown in Table 1. The study included 5,730 participants aged 20–49 years, of whom 1,521 had severe headaches or migraines. The study found that participants with severe headache or migraine were more likely to be female, and participants had higher levels of education, income, and BMI. In addition, the two groups of participants had significant statistical differences in hypertension, smoking status, alcohol consumption, stroke, and HSV infection. The above results were shown in the Table 1.

**Table 1:** Baseline characteristics of the study participants (weighted).

Characteristics	Total	Control	Severe headache or migraine	*P*-value
	*n* = 5,730	*n* = 4,209	*n* = 1,521	
	*N* = 98,044,078	*N* = 72,756,143	*N* = 25,287,934	
Age, years, mean (SD)	34.12 (8.58)	34.16 (8.61)	34.01 (8.50)	0.5
Gender, n (%)				<0.001
Female	3,090 (59)	2025 (45)	1,065 (67)	
Man	2,640 (41)	2,184 (55)	456 (33)	
Race, n (%)				0.2
Mexican American	1,408 (9.1)	1,033 (9.1)	375 (8.9)	
Non-Hispanic Black	290 (6.2)	206 (5.9)	84 (7.2)	
Non-Hispanic White	2,661 (69)	1978 (70)	683 (67)	
Other Hispanic	1,165 (11)	839 (11)	326 (12)	
Other race	206 (4.4)	153 (4.4)	53 (4.4)	
Education, n (%)				<0.001
Below high school	1,400 (16)	977 (14)	423 (20)	
High school	1,398 (26)	1,004 (25)	394 (27)	
Above high school	2,932 (58)	2,228 (60)	704 (53)	
PIR, n (%)				<0.001
≤ 1.3	1,577 (21)	1,041 (18)	536 (29)	
1.3 < PIR < 1.85	754 (11)	538 (10)	216 (12)	
≥ 1.85	3,399 (68)	2,630 (71)	769 (59)	
BMI, n (%)				<0.001
< 25	1974 (37)	1,500 (39)	474 (33)	
25 ≤ BMI < 30	1937 (33)	1,466 (34)	471 (31)	
≥ 30	1819 (30)	1,243 (27)	576 (36)	
Hypertension, n (%)				0.002
No	4,598 (80)	3,437 (81)	1,161 (77)	
Yes	1,132 (20)	772 (19)	360 (23)	
Diabetes, n (%)				0.054
No	5,503 (97)	4,067 (97)	1,436 (96)	
Yes	227 (3.2)	142 (2.9)	85 (4.1)	
Hyperlipidemia, n (%)				0.8
No	3,249 (59)	2,373 (59)	876 (58)	
Yes	2,481 (41)	1836 (41)	645 (42)	
Smoking status, n (%)				0.01
Never	3,224 (53)	2,401 (54)	823 (50)	
Previous smoking	934 (17)	698 (18)	236 (16)	
Current smoking	1,572 (29)	1,110 (28)	462 (33)	
Alcohol consumption, n (%)				<0.001
No	1,602 (24)	1,106 (22)	496 (27)	
Yes	4,128 (76)	3,103 (78)	1,025 (73)	
CVD, n (%)				0.086
No	5,643 (99)	4,158 (99)	1,485 (98)	
Yes	87 (1.5)	51 (1.3)	36 (2.0)	
Stroke, n (%)				0.001
No	5,693 (99)	4,190 (99)	1,503 (98)	
Yes	37 (0.8)	19 (0.5)	18 (1.6)	
HSV-1, n (%)				0.031
No	1901 (39)	1,437 (40)	464 (36)	
Yes	3,829 (61)	2,772 (60)	1,057 (64)	
HSV-2, n (%)				<0.001
No	4,426 (80)	3,333 (82)	1,093 (74)	
Yes	1,304 (20)	876 (18)	428 (26)	
HSV-Subgroup, n (%)				<0.001
HSV-1 (−) and HSV-2 (−)	1,478 (31)	1,151 (33)	327 (26)	
HSV-1 (+) and HSV-2 (−)	2,948 (49)	2,182 (49)	766 (48)	
HSV-1 (−) and HSV-2 (+)	423 (7.9)	286 (7.2)	137 (9.9)	
HSV-1 (+) and HSV-2 (+)	881 (12)	590 (11)	291 (16)	

BMI, body mass index; CVD, Cardiovascular events; PIR, Poverty to income ratio; HSV, Human Simple Virus.

### 3.2 Association between HSV infection and severe headache or migraine

In Model 1 (crude model), we found that HSV-1 (+) participants had an 18% higher risk of severe headache or migraine compared to HSV-1 (−) participants, and HSV-2 (+) participants had a 57% higher risk of severe headache or migraine compared to HSV-2 (−) participants. After adjusting for confounding factors such as sex, age, race, education, and BMI, HSV-2(+) was still significantly associated with severe headache or migraine [(Model 2: OR 1.25, 95%CI:1.04–1.51, *p*-value: 0.0202); (Model 3: OR 1.22, 95%CI:1.03–1.46, *p*-value: 0.0443)]. We divided the participants into four different subgroups based on HSV-1 and HSV-2 infection, and set the HSV-1 (−) and HSV-2 (−) subgroups as the reference group. In Model 1, we found that participants in the HSV-1 (+) and HSV-2 (−) groups, HSV-1 (−) and HSV-2 (+) groups, and HSV-1 (+) and HSV-2 (+) groups had a 22, 72, and 82% higher risk of severe headache or migraine compared to the reference group, respectively. However, after adjusting for confounding factors, only the HSV-1 (−) and HSV-2 (+) groups were still significantly associated with severe headache or migraine [(Model 2: OR 1.42, 95%CI:1.05–1.92, *p*-value: 0.0231); (Model 3: OR 1.41, 95%CI:1.04–1.91, *p*-value: 0.0281)]. All of the above results were shown in Table 2.

**Table 2:** Association of HSV infection with severe headache or migraine in participants of the 1999–2004 NHANES survey.

	Model 1		Model 2		Model 3	
	OR(95% CI)	*P*-value	OR(95% CI)	*P*-value	OR(95% CI)	*P*-value
HSV-1 (+)	1.18(1.01, 1.37)	0.0316	1.05(0.89, 1.24)	0.5809	1.05(0.88, 1.24)	0.5987
HSV-2 (+)	1.57(1.33, 1.85)	0	1.25(1.04, 1.51)	0.0202	1.22(1.03, 1.46)	0.0443
HSV-subgroup						
HSV-1 (−) and HSV-2 (−)	1(Reference)		1(Reference)		1(Reference)	
HSV-1 (+) and HSV-2 (−)	1.22(1.03, 1.46)	0.0246	1.11(0.92, 1.34)	0.2714	1.12(0.92, 1.35)	0.2568
HSV-1 (−) and HSV-2 (+)	1.72(1.30, 2.27)	0.0001	1.42(1.05, 1.92)	0.0231	1.41(1.04, 1.91)	0.0264
HSV-1 (+) and HSV-2 (+)	1.82(1.45, 2.28)	0	1.30(1.00, 1.68)	0.052	1.26(0.96, 1.64)	0.0922

Model 1 is a crude model without adjusting any covariates. Model 2 was adjusted for sex, age, race, education level, poverty to income ratio, and body mass index. Model 3 was based on model 2, and then adjusted for confounding factors such as smoking status, alcohol consumption, hypertension, hyperlipidemia, diabetes, cardiovascular disease and stroke. OR, odds ratio; HSV, human simple virus; CI, confidence interval.

## 4 Discussion

Our study found that being female, higher education, higher BMI, better home conditions, smoking and alcohol consumption were all associated with severe headache or migraine. The difference in the reported incidence of migraine was greatest between the sexes (women vs. men: 17% vs. 8.6%) (1). It may be related to changes in women’s estrogen levels and greater social pressure (16). A higher BMI is a known risk factor for migraine. Obesity can cause migraines through neuropeptides, inflammatory mediators, adipokines, gut microbiota, and changes in eating behavior and lifestyle (17, 18). Alcohol consumption, caffeine intake and smoking are the most common diet-related triggers for increased frequency of migraine attacks (19). In addition, participants with more education and better home conditions may have faced higher expectations and stress, as well as more environmental stimuli, which are likely to trigger migraines.

This study also found that the headache group and the control group had significant statistical differences in hypertension and stroke. Previous studies have shown that people with migraine have a significantly increased risk of hypertension (20, 21). Genome-wide association studies (GWAS) has found that migraine and blood pressure share common genetic loci, and cross-trait association analysis have revealed potential common biological mechanisms between migraine and blood pressure regulation, such as vascular development, endothelial function, and neurogenic inflammation (22). In addition, there is a lot of evidence that migraines increase the risk of stroke (23). Peng et al. found that patients with migraine had a 24% higher risk of ischemic stroke compared to the control group (24). In Lee’s study, patients with migraine had an 18% higher risk of ischemic stroke (25). The pathological mechanism of migraine leading to stroke may be related to cortical diffusion inhibition, endothelial dysfunction, microembolism, coagulation dysfunction, etc. (26, 27).

At present, there are few studies on HSV infection and migraine. Napier et al. reported that after 1 year of using famciclovir, patients’ migraine symptoms were significantly reduced (6). In their retrospective clinical study, Meineri et al. found that 42% of patients with new daily persistent headaches had recently been infected with HSV (7). Our findings suggest that HSV infection is positively associated with severe headache or migraine, especially HSV-2 infection. In subgroup analyses, we also found that participants with HSV-1 (+) and HSV-2 (+), HSV-1 (−) and HSV-2 (+) had a 50 and 60% higher risk of severe headaches or migraines than participants with HSV-1 (+) and HSV-2 (−), respectively. In addition, after we adjusted for potential confounding factors, only participants with HSV-2 (+), participants with HSV-1 (−) and HSV-2 (+) were also significantly associated with severe headaches or migraines. Both HSV-1 and HSV-2 are known to be latent in the trigeminal ganglion (28, 29). The trigeminal ganglion is a major component of the trigeminal nerve, which plays an important role in the pathogenesis of migraine. The trigeminal ganglion contains neurons that transmit sensations such as pain, touch, and pressure. KCNK18, a migraine susceptibility gene, encodes neuronal potassium channels that are highly enriched in the trigeminal ganglion (30). Previous studies have shown that dominant negative mutations in this gene cause a loss of channel conductance, leading to familial migraine (31, 32). However, it is unclear whether HSV can cause migraines by causing mutations in the KCNK18 gene. HSV infection is closely related to neuroinflammation. When the virus is activated, host immune cells such as microglia and astrocytes are activated, releasing inflammatory mediators such as cytokines and chemokines, exacerbating neuroinflammation (33). Neuroinflammation plays an important role in the pathophysiology of migraine. For example, the activation of inflammatory bodies can lead to the production of inflammatory cytokines, which stimulate trigeminal neurons and cause migraines (34). In the pathophysiology of migraine, neurogenic inflammation and neuroinflammation can affect the dural vessels, trigeminal nerve endings, trigeminal ganglion, trigeminal caudal nucleus, central trigeminal nerve pain processing structures (35). HSV infection may lead to migraine or headache by causing neuroinflammation, but the exact pathophysiological mechanism needs to be further explored.

In Model 1 (crude model), HSV-1 (+) is associated with headaches or migraines. However, after adjusting for potential confounding factors, HSV-1 (+) was not significantly associated with headaches or migraines compared to HSV-2 (+). Both HSV-1 and HSV-2 can cause oral and genital herpes (36, 37). In general, the symptoms of genital herpes are usually more severe than those of oral cold sores. Clinical symptoms of primary genital herpes often include headache, fever, muscle pain, etc. (37). In the past, it was thought that 90% of genital herpes was caused by HSV-2 infection, but with the change of sexual behavior, the proportion of HSV-1 infection caused by oral genital sexual behavior has increased significantly (10% ~ 40%) (38). After genital infection, T cells control the colonization of sensory ganglia by HSV-2 less efficiently than HSV-1 (39). The recurrence frequency and aggressiveness of HSV-2 are also higher than that of HSV-1 (40). HSV-2 is the main cause of recurrent genital herpes infections. Its frequent recurrence, may bring lasting pain to the patient, greater psychological pressure, and thus more likely to induce migraine. Studies have shown that HSV-2 can promote neurite growth by enhancing the activity of nerve growth factor (41, 42). Abnormal growth or regeneration of neurites is associated with neuroinflammation, neurotransmitter release, or abnormal excitability of the nervous system. These may all be related to an overreaction of the central nervous system to pain signals. In addition, although nerve growth factor can improve nerve regeneration, it can also accelerate neuropathic pain (43, 44). Reactivation of HSV-2 may lead to sacral root neuritis. Neuroinflammation caused by sacral root neuritis may spread to the head via nerve pathways, causing headaches. In addition, sacral radicular neuritis can cause chronic pain and may produce significant stress and anxiety, which are also predisposing factors for migraines. Neuroinflammation caused by sacral root neuritis may spread to the head via nerve pathways, causing headaches. In addition, sacral radicular neuritis can cause chronic pain and may produce significant stress and anxiety, which are also predisposing factors for migraines.

There are some limitations to our study. First, although our study accounted for a number of potential confounding factors, we cannot rule out the influence of other factors on the results, such as the overall health of the patients, co-existing infections, or environmental influences. Second, although we found an association between HSV infection and migraine or headache, we cannot be sure whether there is a causal relationship. Third, our study population was participants aged 20–49 years in the United States, and the results may not be applicable to all populations, or other ethnicities.

## 5 Conclusion

HSV-2 gG-2(+) was significantly associated with severe headache or migraine.

## Data Availability

The original contributions presented in the study are included in the article/supplementary material, further inquiries can be directed to the corresponding authors.

## Footnotes

^1^ https://www.statsape.com/
